# The relationship between inflammatory cytokines and in‐hospital complications of acute pancreatitis

**DOI:** 10.1002/iid3.1203

**Published:** 2024-02-27

**Authors:** Jiaxin Yao, Shuangshuang Zhang, Fei Zhou, Mengting Zhuang, Sujuan Fei

**Affiliations:** ^1^ Department of Gastroenterology Xuzhou Medical University Xuzhou China; ^2^ Department of Gastroenterology The Affiliated Hospital of Xuzhou Medical University Xuzhou China; ^3^ Key Laboratory of Gastrointestinal Endoscopy Xuzhou Medical University Xuzhou China

**Keywords:** acute necrotic collection, acute pancreatitis, acute peripancreatic fluid collection, ascites, cytokines, pleural effusion

## Abstract

**Objective:**

Acute necrotic collection (ANC), acute peripancreatic fluid collection (APFC), pleural effusion, and ascites are common early complications of acute pancreatitis. This study aimed to investigate the relationship between 12 serum cytokines and the early complications and severity of acute pancreatitis (AP).

**Methods:**

We retrospectively analyzed the clinical data of 307 patients with AP, and divided them into severe group and mild‐to‐moderate group according to the revised Atlanta classification. Propensity score matching was used to control for confounding factors. Binary logistic regression analysis was used to explore the relationship between cytokine levels and early complications of AP.

**Results:**

Serum levels of interleukin (IL)‐1β, IL‐5, IL‐6, IL‐8, IL‐10, IL‐17, and tumor necrosis factor‐α were significantly higher in the severe acute pancreatitis (SAP) group than in the non‐SAP group (*p* < .05). After adjusting for confounding factors, the upper quartiles of IL‐6, IL‐8, and IL‐10 were associated with an increased risk of ANC compared with those in the lowest quartile (IL‐6: quartile 3, odds ratio [OR] = 3.99, 95% confidence interval [CI] = 1.95–8.16; IL‐8: quartile 4, OR = 2.47, 95% CI = 1.27–4.84; IL‐10: quartile 2, OR = 2.22, 95% CI = 1.09–4.56). APFC was associated with high serum levels of IL‐6 (quartile 3, OR = 1.32, 95% CI = 1.02–1.72), pleural effusions were associated with high serum levels of IL‐1β, IL‐6, IL‐8, and IL‐10 (IL‐1β: quartile 4, OR = 2.36, 95% CI = 1.21–4.58; IL‐6: quartile 3, OR = 4.67, 95% CI = 2.27–9.61; IL‐8: quartile 3, OR = 2.95, 95% CI = 1.51–5.79; IL‐10: quartile 4, OR = 3.20, 95% CI = 1.61–6.36), and high serum levels of IL‐6 and IL‐10 were associated with an increased risk of ascites (IL‐6: quartile 3, OR = 3.01, 95% CI = 1.42–6.37; IL‐10: quartile 3, OR = 2.57, 95% CI = 1.23–5.37).

**Conclusion:**

Serum cytokine levels, including IL‐1β, IL‐6, IL‐8, and IL‐10 may be associated with the occurrence of early complications of AP. In daily clinical practice, IL‐6 may be the most worthwhile cytokine to be detected.

## Graphical abstract

Acute necrotic collection, acute peripancreatic fluid collection, pleural effusion, and ascites are common early complications of acute pancreatitis (AP). This study aimed to investigate the relationship between 12 cytokines and the early complications and severity of AP.

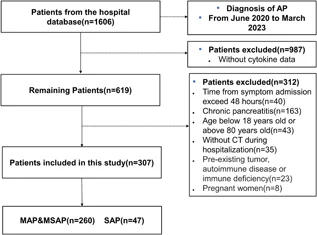

## 1 INTRODUCTION

Acute pancreatitis (AP) is one of the most common gastrointestinal diseases, with a variable course that is often difficult to predict in its early stages.^1^ About 80% of patients develop mild to moderately severe disease. However, about 20% of patients develop severe acute pancreatitis (SAP), with serious complications and a mortality rate of 20%–30%.^2^ Gallstones, hypertriglyceridemia, and alcohol abuse are the most common causes of AP. In China, the proportion of hypertriglyceridemia (HTG)‐related pancreatitis is increasing year by year. Uncommon causes include drugs, endoscopic retrograde cholangiopancreatography (ERCP), hypercalcemia, infections, genetics, autoimmune diseases, and (surgical) trauma.^3^

So far, SAP remains challenging in contemporary intensive care due to its unpredictable development and high mortality. SAP often accompanies severe complications, and the occurrence of complications is often an important reason for prolonging the course of disease, increasing hospitalization time and mortality.^4^ The complications of AP can be local or distant, immediate or delayed. Interstitial edematous pancreatitis and necrotizing pancreatitis are the main two subtypes of AP. Interstitial edematous pancreatitis is characterized by inflammation and edema of the pancreatic parenchyma and peripancreatic tissues. Necrotizing pancreatitis occurs when this process progresses to cell death. Acute peripancreatic fluid collection (APFC) is a homogeneous fluid located within or near the pancreas, lacking a clear wall, occurring in the early course of AP, mainly within 4 weeks of onset. However, acute necrotic collection (ANC) occurs in necrotizing pancreatitis, consisting of necrotic material and liquid phase components. APFC and ANC that persist after 4 weeks of onset are respectively called pancreatic pseudocyst (PPC) and walled‐off necrosis.^5^ However, in addition to the common local complications of the pancreas, pulmonary complications are an inevitable topic for AP patients. Pleural effusion is one of the common pulmonary complications in AP patients. The latest data show that the incidence of AP combined with pleural effusion is as high as 34%–54.5%.^6^, ^7^ The potential mechanisms of pleural effusion accumulation in AP are diverse and can be explained by the following reasons: inflammation‐induced changes in capillary permeability, diaphragmatic lymphatic obstruction, pleural‐pancreatic fistula formation, and sinus formation between the pleural cavity and PPC.^8^ Furthermore, up to 30%–40% of AP patients develop ascites, and a large proportion of patients develop mild to moderate ascites in the initial stage of inflammation, which may be secondary to local inflammation and subsequent transperitoneal and vascular exudation.^9^, ^10^ In the late stage of AP, ascites are rare, usually due to pancreatic necrosis leading to pancreatic duct rupture. In patients with SAP, if clinically significant intra‐abdominal hypertension occurs, early drainage of ascites may be required, and up to 60%–80% of patients with SAP may develop ascites.^11^

Early complications of AP, as key events of disease prognosis, are crucial for AP patients to be identified and intervened early.^12^, ^13^ However, to our knowledge, only limited clinical studies have focused on the relationship between some serological indicators (such as C‐reactive protein [CRP], neutrophil‐to‐lymphocyte ratio [NLR], etc.) and early complications of AP, and hardly any studies have systematically evaluated the relationship between cytokine profiles and early complications of AP.^14^ The activation of the immune system has been identified as a key trigger and regulator of pancreatitis‐induced injury. The uncontrolled activation of inflammatory signals and the intense release of inflammatory cytokines resulted in the severity of the patients.^15^ Researchers have found that inflammation can spread from the local pancreas to nearby tissues, or cause systemic inflammation through the cascade activation of cytokines.^16^, ^17^, ^18^ In this context, We retrospectively analyzed the data of 12 serum cytokines at admission of 307 patients with AP, and further evaluated their relationship with AP severity and early complications.

## 2 MATERIALS AND METHODS

### 2.1 Population

This retrospective study collected information from 1606 patients with AP admitted to the Affiliated Hospital of Xuzhou Medical University from June 2020 to March 2023. The study was conducted in accordance with the principles of the Declaration of Helsinki and was approved by the local ethics committee (Clinical Research Professional Ethics Committee of the First Affiliated Hospital of Xuzhou Medical University, China). Approval number: XYFY2023‐KL083‐01.

### 2.2 The inclusion criteria

(1) First diagnosed with AP. (2) Age of at least 18 and not more than 80 years. (3) Patients with test results of 12 cytokines drawn within 48 h of symptom onset. (4) Chest (nonenhanced) and abdominal (nonenhanced and enhanced) computed tomography examination.

### 2.3 The exclusion criteria

(1) Chronic pancreatitis. (2) Long‐term immunomodulatory therapy, including corticosteroids. (3) Severe cardiovascular or pulmonary disease, acquired immunodeficiency syndrome, autoimmune diseases, malignant tumors. (4) Pregnant patients. (5) Patients with incomplete clinical data.

**Figure 1:**
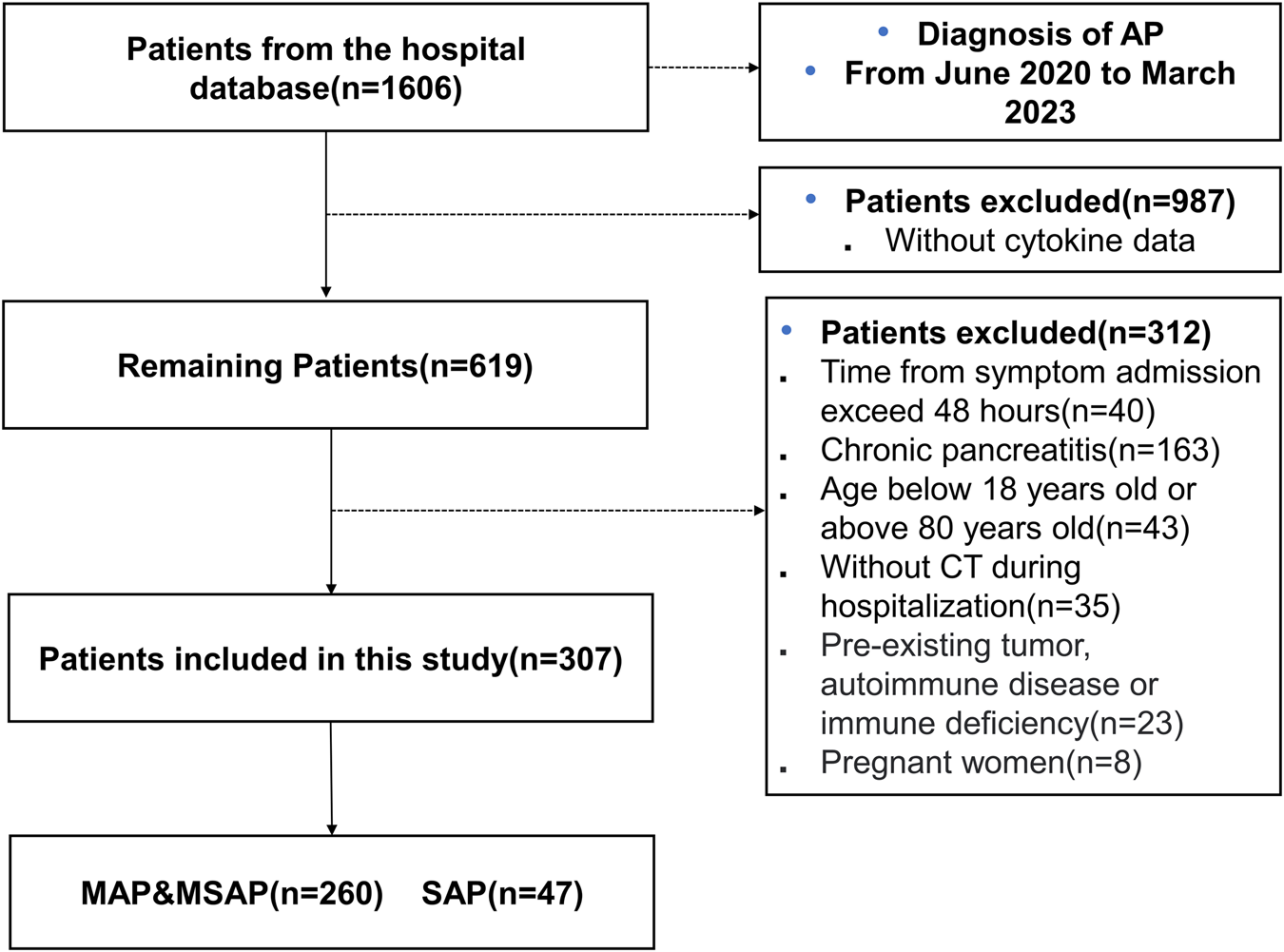
Flow chart. AP, acute pancreatitis; CT, computed tomography; MAP, mild acute pancreatitis; MSAP, moderately severe acute pancreatitis; SAP, severe acute pancreatitis.

Finally, 307 patients were confirmed to be included in this study, while 1299 patients were excluded due to the exclusion criteria (Figure 1).

### 2.4 Grouping criteria

According to the revised Atlanta classification in 2012: (1) Mild acute pancreatitis (MAP), without organ dysfunction and local or systemic complications. (2) Moderately severe acute pancreatitis (MSAP), with transient (≤48 h) organ dysfunction and/or local complications. (3) SAP, with persistent (>48 h) organ dysfunction. The diagnosis of organ dysfunction was based on the modified Marshall scoring system, and any organ score ≥2 points could define the presence of organ dysfunction. Finally, all patients were divided into two groups: severe group and mild to moderate group.

### 2.5 Laboratory tests

Laboratory tests included white blood cell (WBC) count, neutrophils, lymphocytes, and serum cytokines, including interleukin (IL)‐1β, IL‐2, IL‐4, IL‐5, IL‐6, IL‐8, IL‐10, IL‐12p70, IL‐17, interferon (IFN)‐γ, IFN‐α, tumor necrosis factor (TNF)‐α. The routine blood parameters of the patients were measured by Sysmex XN 2000 automatic hematology analyzer, the blood biochemical parameters were measured by Cobas 8000 automatic biochemical immunoassay analyzer and its matching reagents, the serum inflammatory factors were measured by Beckman Navios flow cytometer, and the reagent kit was 12 cytokine detection kit (multiplex microsphere flow immunofluorescence method; Qingdao Ruisikai Biotechnology Co., Ltd.); the patients' elbow venous blood was collected and sent for testing immediately within 60 min after collection, and the testing was performed by laboratory doctors.

### 2.6 Data analysis

Statistical analysis was performed using SPSS 26.0 and R (R Core Team, 2021). R: A language and environment for statistical computing. R Foundation for Statistical Computing. URL https://www.R-project.org/) and RStudio (RStudio Team, 2022). RStudio: Integrated Development Environment for R. RStudio, PBC, URL http://www.rstudio.com/). The normality of continuous data was assessed by the Kolmogorov–Smirnov test. For nonnormally distributed variables, data were presented as median (interquartile range). Group differences of continuous data were tested by the Mann–Whitney *U* test. For categorical variables, group differences were tested by the *χ* ^2^ square test and Fisher's exact test.

The predictive ability of cytokines for AP severity was defined by the area under the curve (AUC) in the receiver operating characteristic (ROC) analysis. The optimal cut‐off value was based on the maximum Youden index to discriminate sensitivity and specificity. Cytokines as continuous variables (per 1‐SD augment) and ordinal categorical variables (based on quartiles, Q1, Q2, Q3, Q4) were used to determine the relationship between cytokines and the presence and adjusted presence of ANC, APFC, pleural effusion, and ascites by binary logistic regression analysis. Odds ratios (ORs) and 95% confidence intervals (CIs) were calculated. We first performed a crude logistic regression analysis to establish Model 1, to examine the unadjusted association between cytokines and outcomes. Next, to eliminate the potential bias caused by confounding factors, we established two multivariate logistic regression models, Model 2 adjusted for age, gender, and body mass index (BMI); and Model 3 for past medical history, to obtain adjusted ORs and 95% CIs. Finally, we performed a sensitivity analysis by propensity score matching (PSM), PSM used 1:2 matching (using 1:2 nearest neighbor matching method, caliper value of 0.15), and the covariates entered into the PSM model were: age, gender, BMI, and past medical history, to re‐evaluate the relationship between cytokines and complications.

## 3 RESULTS

### 3.1 Population characteristics

A total of 307 patients with AP were enrolled, with a median age of 45 years. Among these, 144 (46.7%) were males and 163 (53.1%) were females, 115 (37.5%) were diagnosed with MAP, 145 (47.2%) with MSAP, and 47 (15.3%) with SAP. There was no statistically significant difference in gender and age between the two groups, respectively (*p* = .925, *p* = .516). For the etiology of AP, there were 94 cases (30.6%) of cholelithiasis, 125 cases (40.7%) of HTG, 27 cases (8.8%) of alcoholism, and 13 cases (4.2%) of post‐ERCP. In addition, there were three cases (1%) of trauma and 45 cases (14.7%) of autoimmune and unknown (idiopathic) nature. The baseline characteristics and etiology of the 307 patients with AP are shown in Table 1. The severe group had higher levels of WBC count, neutrophil count, lymphocyte count, NLR, and CRP than the non‐SAP group, and lower blood calcium levels than the mild‐to‐moderate group (*p* < .001). The SAP group had a higher probability of developing necrosis, pleural effusion, ascites, organ failure and other related complications, admission to the intensive care unit (ICU), and length of hospital stay than the mild‐to‐moderate group (*p* < .001).

**Table 1:** Demographic data and clinical characteristics of 307 AP patients.

Characteristics	All patients	MAP and MSAP (*n* = 260)	SAP (*n* = 47)	*p*‐Value
Patient characteristics				
Age, median (IQR), years	45 (34, 63)	45 (34, 60.75)	44 (33, 70)	.925
Male, *n* (%)	144 (46.9)	124 (47.7)	20 (42.6)	.516
BMI, median (IQR)	26.1 (23.9, 28.1)	26.1 (23.9, 28.7)	26 (23.8, 26.2)	.148
History				
Hypertension, *n* (%)	98 (31.9)	75 (28.8)	23 (48.9)	.007
Diabetes mellitus, *n* (%)	91 (29.6)	71 (27.3)	20 (42.6)	.035
Coronary heart disease, *n* (%)	22 (7.2)	15 (5.8)	7 (14.9)	.026
Smoking, *n* (%)	68 (22.1)	63 (24.2)	5 (10.6)	.039
Drinking, *n* (%)	46 (15)	41 (15.8)	5 (10.6)	.364
Cause of AP, *n* (%)				
Gallstone	94 (30.6)	80 (30.8)	14 (29.8)	
Hyperlipidemia	125 (40.7)	107 (41.2)	18 (38.3)	
Alcohol abuse	27 (8.8)	26 (10.0)	1 (2.1)	
Post‐ERCP	13 (4.2)	10 (3.8)	3 (6.4)	
Trauma	3 (1.0)		3 (6.4)	
Other	45 (14.7)	37 (14.2)	8 (17.0)	
Clinical outcomes				
Duration of hospitalization, median (IQR), days	9 (6, 13)	8 (6, 11)	18 (11, 24)	<.001
ICU admission, *n* (%)	25 (8.1)	2 (0.8)	23 (48.9)	<.001
ANC, *n* (%)	119 (38.8)	81 (31.2)	38 (80.9)	<.001
APFC, *n* (%)	50 (16.3)	33 (12.7)	17 (36.2)	<.001
Pleural effusion, *n* (%)	134 (43.6)	91 (35.0)	43 (91.5)	<.001
Ascites, *n* (%)	105 (34.2)	71 (27.3)	34 (72.3)	<.001
SIRS, *n* (%)	67 (21.8)	34 (13.1)	33 (70.2)	<.001
Organ failure, *n* (%)	69 (22.5)	22 (8.5)	47 (100)	<.001
Death, *n* (%)	1 (0.3)		1 (0.3)	.153
Laboratory parameters				
WBC (×109/L), median (IQR)	11.7 (8.2, 15.3)	11.3 (8, 14.5)	14.4 (10.9, 18.8)	.001
Neutrophil count (×109/L), median (IQR)	9.5 (6, 13)	9 (5.8, 12.3)	13 (8.8, 17.1)	<.001
Lymphocyte count (×109/L), median (IQR)	1.1 (0.8, 1.6)	0.8 (1.2, 1.7)	0.9 (0.6, 1.3)	.002
NLR, median (IQR)	8.4 (4.7, 14)	7.5 (4.4, 12)	15.6 (8.8, 20.9)	<.001
CRP (mg/L), median (IQR)	60.8 (8.4, 164.1)	40.7 (6.4, 136.7)	168 (81.5, 252.8)	.001
Calcium (mmol/L), median (IQR)	2.11 (1.95, 2.22)	2.13 (2.0, 2.23)	1.89 (1.13, 2.16)	<.001

Abbreviations: ANC, acute necrotic collection; APFC, acute peripancreatic fluid collection; BMI, body mass index; CRP, C‐reactive protein; ERCP, endoscopic retrograde cholangiopancreatography; ICU, intensive care unit; IQR, interquartile range; MAP, mild acute pancreatitis; MSAP, moderately severe acute pancreatitis; NLR, neutrophil‐to‐lymphocyte ratio; SAP, severe acute pancreatitis; SIRS, systemic inflammatory response syndrome; WBC, white blood cell count.

### 3.2 The relationship between cytokine levels and the severity AP

**Figure 2:**
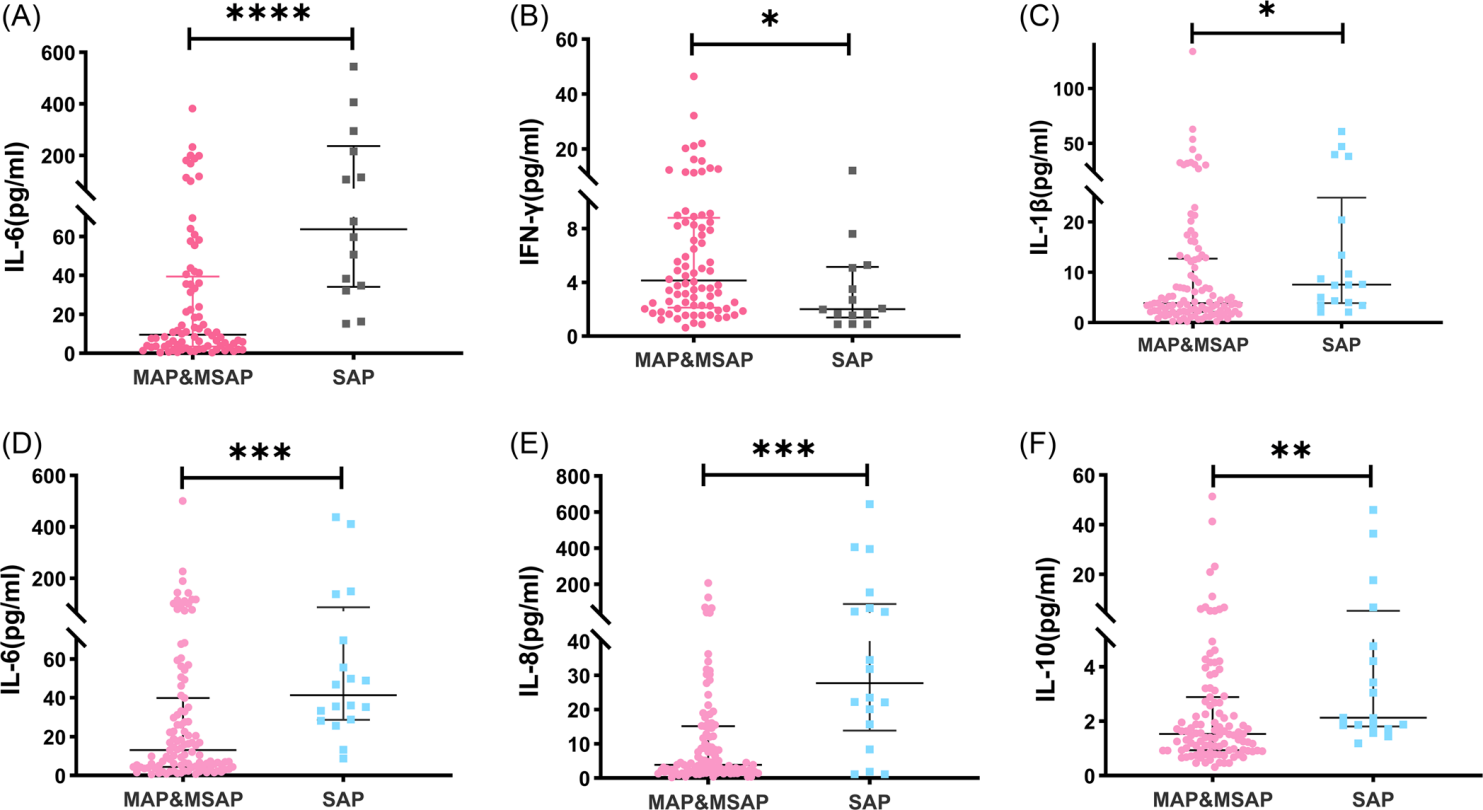
Scatter plot of the cytokines with statistical significance between the MAP and MSAP group and the SAP group under different etiologies. (A, B) Cholelithiasis group. (C–F) Hyperlipidemia group. IFN‐γ, interferon‐gamma; IL‐1β, interleukin‐1βeta; IL‐6, interleukin‐6; IL‐8, interleukin‐8; IL‐10, interleukin‐10; MAP, mild acute pancreatitis; MSAP, moderately severe acute pancreatitis; SAP, severe acute pancreatitis.

By using nonparametric tests to compare the differences in cytokines between the mild‐to‐moderate group and the SAP group (Table 2), the serum levels of IL‐1β, IL‐5, IL‐6, IL‐8, IL‐10, IL‐17, and TNF‐α in SAP group were significantly higher than those in the mild‐to‐moderate group (*p* < .05). Among them, the differences of IL‐6, IL‐8, and IL‐10 between the two groups were very significant (*p* < .001). In addition, hyperlipidemia was the most prevalent etiology in this study, followed by cholelithiasis. In cholelithiasis, compared with the non‐SAP group, the SAP group had significant differences in IL‐6 and IFN‐γ (*p* < .05), however, IL‐1β, IL‐5, IL‐8, IL‐10, IL‐17, TNF‐α did not show statistical differences between the two groups. In hyperlipidemia, IL‐1β, IL‐6, IL‐8, and IL‐10 still had significant differences between the SAP group and non‐SAP group (*p* < .05), while IL‐5, IL‐17, TNF‐α had no statistical differences between the two groups (Figure 2).

**Table 2:** Comparison of cytokine levels between the MAP and MSAP group and the SAP group.

Inflammatory cytokines (pg/mL)	MAP and MSAP (*n* = 260)	SAP (*n* = 47)	*p*‐Value
IL‐1β	4.49 (2.31–12.44)	7.59 (3.09–23.09)	.023
IL‐2	1.94 (1.09–9.08)	2.34 (1.72–10.82)	.075
IL‐4	1.41 (0.98–3.20)	1.91 (1.22–4.00)	.272
IL‐5	2.00 (1.36–3.74)	3.42 (1.35–7.04)	.029
IL‐6	11.06 (3.88–38.00)	48.91 (32.73–114.09)	<.001
IL‐8	4.46 (2.06–15.91)	21.65 (8.44–67.72)	<.001
IL‐10	1.66 (0.94–2.92)	2.29 (1.72–6.89)	<.001
IL‐12p70	1.60 (1.10–2.61)	1.96 (1.44–2.67)	.111
IL‐17	1.79 (1.13–3.30)	2.42 (1.34–4.20)	.037
IFN‐α	1.68 (0.95–3.21)	2.10 (1.08–5.99)	.121
IFN‐γ	3.58 (1.98–7.16)	2.99 (1.63–8.25)	.406
TNF‐α	1.92 (1.39–3.24)	2.49 (1.54–4.84)	.041

Abbreviations: IFN‐α, interferon‐alpha; IFN‐γ, interferon‐gamma; IL‐1β, interleukin‐1βeta; IL‐2, interleukin‐2; IL‐4, interleukin‐4; IL‐5, interleukin‐5; IL‐6, interleukin‐6; IL‐8, interleukin‐8; IL‐10,interleukin‐10; IL‐12p70, interleukin‐12p70; IL‐17, interleukin‐17; MAP, mild acute pancreatitis; MSAP, moderately severe acute pancreatitis; SAP, severe acute pancreatitis; TNF‐α, tumor necrosis factor‐alpha.

### 3.3 The diagnostic value of cytokines in predicting SAP

We further used the ROC curves of cytokines to predict SAP and obtained their respective cut‐off values and evaluation abilities. The results showed that the AUCs of IL‐1β, IL‐5, IL‐6, IL‐8, IL‐10, IL‐17, and TNF‐α for assessing SAP were 0.604, 0.600, 0.788, 0.721, 0.676, 0.596, 0.594, respectively. Their optimal diagnostic cut‐off values were 7.42, 3.09, 24.67, 8.07, 1.72, 1.95, and 2.35 pg/mL, respectively (Figure S1 and Table 3).

**Table 3:** Prognostic value of cytokines for predicting SAP.

Inflammatory cytokines	Cut‐off	Sensitivity (%)	Specificity (%)	AUC	*p*‐Value	95% CI
IL‐1β	7.42	55.32	66.15	0.604	.0232	0.5132–0.6948
IL‐5	3.09	57.45	68.08	0.6004	.0285	0.5052–0.6955
IL‐6	24.67	87.23	66.92	0.7875	<.001	0.7280–0.8470
IL‐8	8.07	80.85	61.92	0.7207	<.001	0.6365–0.8050
IL‐10	1.72	76.60	52.31	0.6763	<.001	0.5970–0.7556
IL‐17	1.95	63.83	60.38	0.5957	.0367	0.5092–0.6823
TNF‐α	2.345	55.32	61.92	0.5936	.0412	0.5066–0.6806

Abbreviations: 95% CI, 95% confidence interval; AUC, area under the ROC curve; IL‐1β, interleukin‐1βeta; IL‐5, interleukin‐5; IL‐6, interleukin‐6; IL‐8, interleukin‐8; IL‐10, interleukin‐10; IL‐17, interleukin‐17; TNF‐α, tumor necrosis factor‐alpha.

### 3.4 The relationship between cytokine levels and AP complications

**Figure 3:**
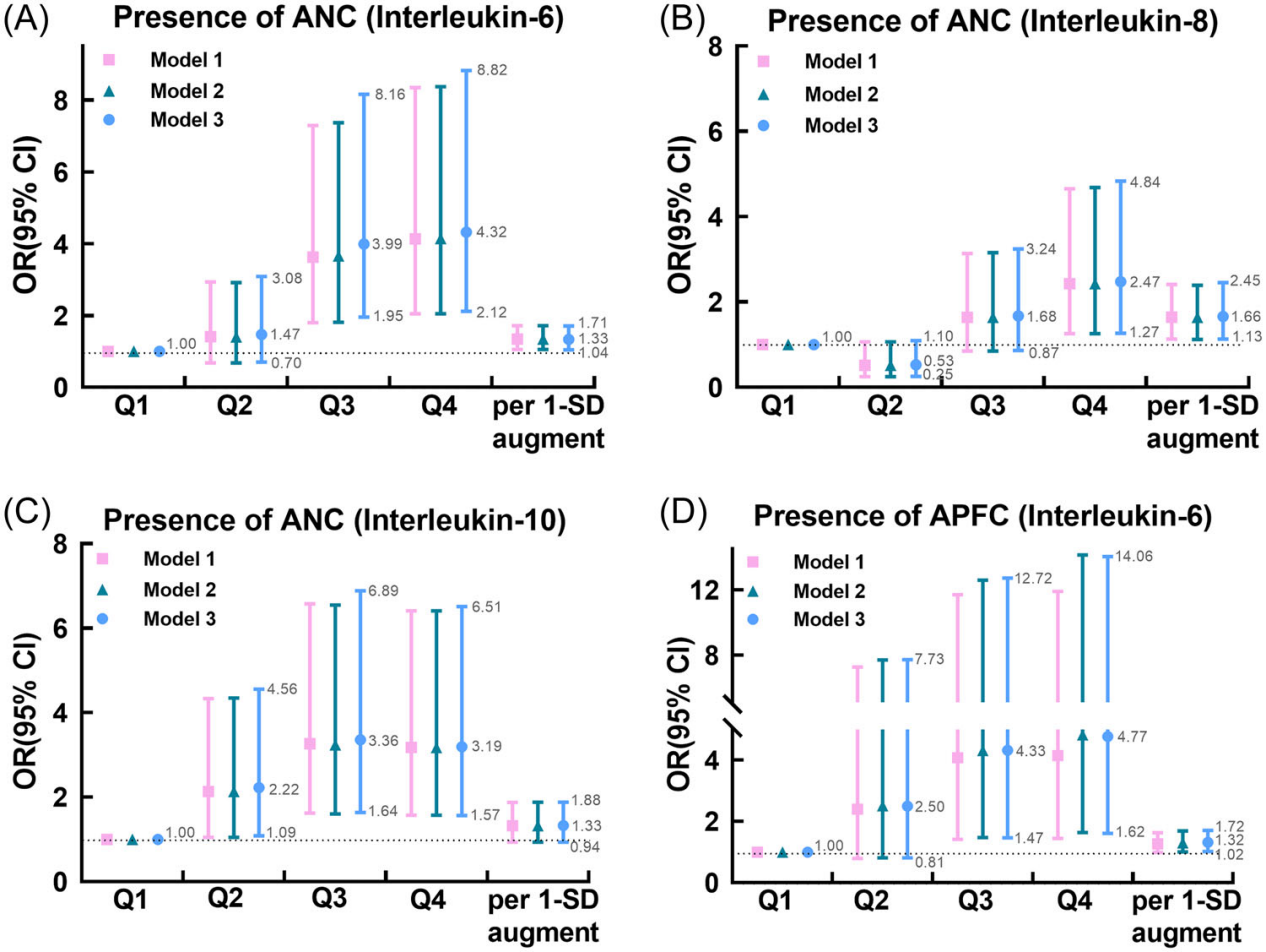
The serum cytokine levels in association with the presence of ANC and APFC. Forest plots present ORs/cORs for the associations of the serum IL‐6 levels with the presence of ANC (A), the serum IL‐8 levels with the presence of ANC (B), the serum IL‐10 levels with the presence of ANC (C) and the serum IL‐6 levels with the presence of APFC (D). The colorful lines indicate 95% CIs of ORs/cORs. Model 1—crude logistic regression analysis; Model 2—adjusted for age, gender, and BMI; Model 3—past medical history (hypertension, diabetes mellitus, coronary heart disease, smoking, drinking). IL‐6 (Q1 ≤ 4.47, Q2: 4.48–15.18, Q3: 15.19–48.91, Q4 ≥ 48.92); IL‐8 (Q1 ≤ 2.30, Q2: 2.31–6.72, Q3: 6.73–19.53, Q4 ≥ 19.54); IL‐10 (Q1 ≤ 1.05, Q2: 1.06–1.75, Q3: 1.76–3.38, Q4 ≥ 3.39). ANC, acute necrotic collection; APFC, acute peripancreatic fluid collection; BMI, body mass index; CI, confidence interval; cOR, crude odds ratio; IL‐6, interleukin‐6; IL‐8, interleukin‐8; IL‐10, interleukin‐10; OR, odds ratio.

High cytokine levels were closely related to the occurrence of ANC, APFC, pleural effusion, and ascites. In the three models, we compared the variables of the higher three quartiles with the first quartile and listed the ORs and 95% CIs corresponding to the second, third, and fourth quartiles. In Model 3, which adjusted for all potential confounding variables, compared with the lowest quartile, the third quartile (Q3) of IL‐6 reflected an increased risk of ANC (OR = 3.99, 95% CI = 1.95–8.16, *p* < .001), the highest quartile (Q4) of IL‐8 reflected an increased risk of necrosis (OR = 2.47, 95% CI = 1.27–4.84, *p* < .01), and the second quartile (Q2) of IL‐10 reflected an increased risk of necrosis (OR = 2.22, 95% CI = 1.09–4.56, *p* < .05). Similarly, IL‐6 and IL‐8 showed similar results (*p* < .05) when analyzed in the continuous model (per 1‐SD augment). However, IL‐10 was not statistically significant when analyzed in the continuous model (per 1‐SD augment). Only IL‐6 showed an increased risk of APFC in the third quartile (Q3) (OR = 1.32, 95% CI = 1.02–1.72, *p* < .01), and remained statistically significant in the continuous model analysis (per 1‐SD augment) (*p* < .05), while the other cytokines did not show predictive value for APFC (Figure 3 and Table S1).

**Figure 4:**
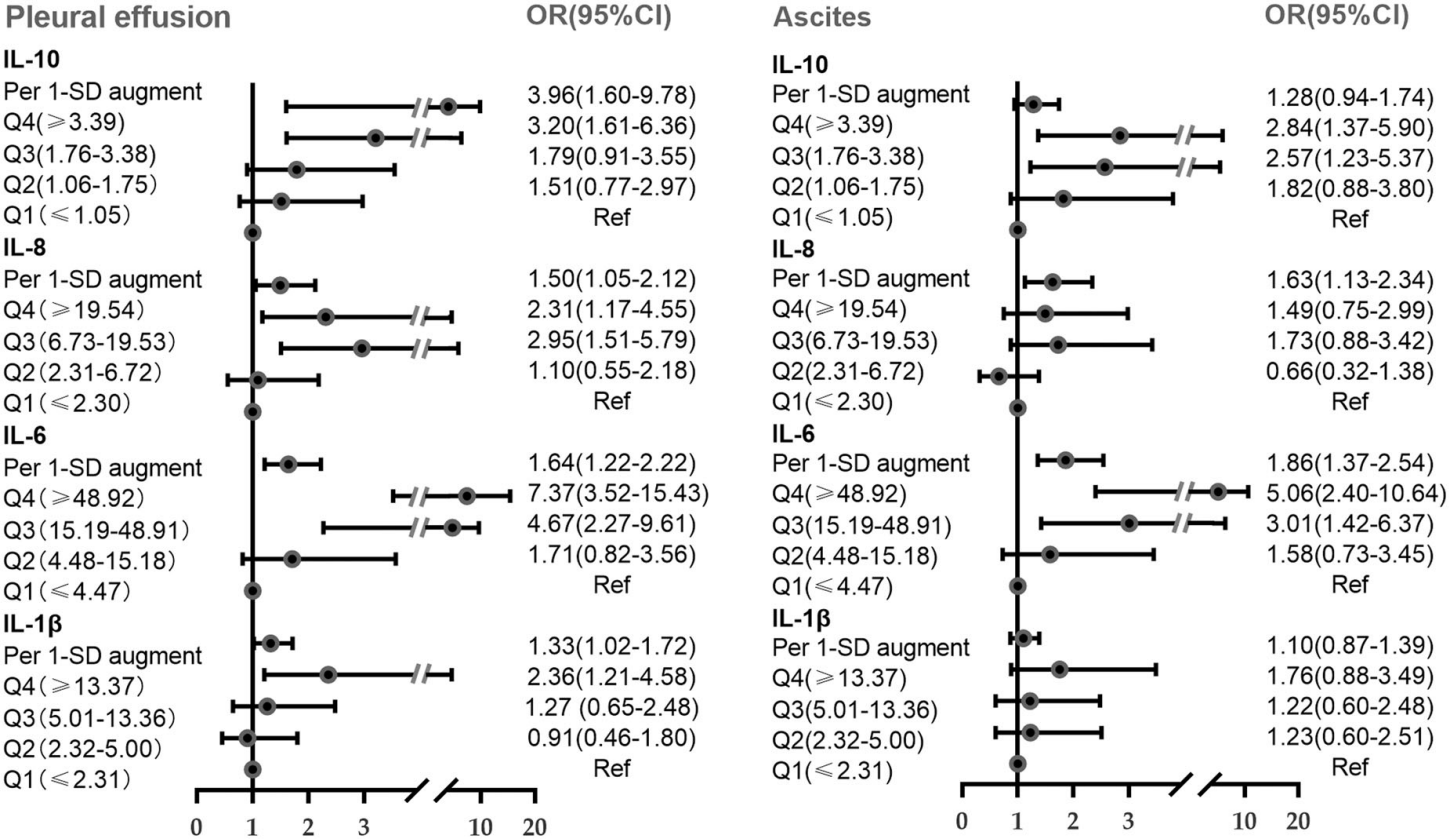
The serum cytokine levels in association with the presence of pleural effusion and ascites. Forest plots present ORs for the associations of the serum cytokine levels with the presence of pleural effusion and ascites. The black lines indicate 95% CIs of ORs. Multivariable logistic regression model adjusted for all potential confounders. IL‐1β (Q1 ≤ 2.31, Q2: 2.32–5.00, Q3: 5.01–13.36, Q4 ≥ 13.37); IL‐6 (Q1 ≤ 4.47, Q2: 4.48–15.18, Q3: 15.19–48.91, Q4 ≥ 48.92); IL‐8 (Q1 ≤ 2.30, Q2: 2.31–6.72, Q3: 6.73–19.53, Q4 ≥ 19.54); IL‐10 (Q1 ≤ 1.05, Q2: 1.06–1.75, Q3: 1.76–3.38, Q4 ≥ 3.39). CI, confidence interval; IL‐1β, interleukin‐1βeta; IL‐6, interleukin‐6; IL‐8, interleukin‐8; IL‐10, interleukin‐10; OR, odds ratio.

In Model 3, when the outcome variable was pleural effusion, compared with the lowest quartile (Q1), the highest quartile (Q4) of IL‐1β reflected an increased risk of pleural effusion (OR = 2.36, 95% CI = 1.21–4.58, *p* < .05), the third quartile (Q3) of IL‐6 reflected an increased risk of pleural effusion (OR = 4.67, 95% CI = 2.27–9.61, *p* < .001), the third quartile (Q3) of IL‐8 reflected an increased risk of pleural effusion (OR = 2.95, 95% CI = 1.51–5.79, *p* < .01), and the highest quartile (Q4) of IL‐10 reflected an increased risk of pleural effusion (OR = 3.20, 95% CI = 1.61–6.36, *p* < .01). When analyzed in the continuous model (per 1‐SD augment), IL‐1β, IL‐6, IL‐8, and IL‐10 also showed similar results (*p* < .05) (Figure 4 and Table S2).

When the outcome variable was ascites, Model 3, IL‐6 was associated with an increased risk of ascites in the third quartile (Q3) compared with the lowest quartile (Q1) (OR = 3.01, 95% CI = 1.42–6.37, *p* < .01), the third quartile (Q3) of IL‐10 reflected an increased risk of ascites (OR = 2.57, 95% CI = 1.23–5.37, *p* < .05), IL‐1β and IL‐8 results were not statistically significant. IL‐6 and IL‐8 reflected an increased risk of ascites when analyzed in the continuous model (per 1‐SD augment) (*p* < .01) (Figure 4 and Table S3).

To reduce data bias and confounding variables, patients with complications and those without complications were matched at a 1:2 ratio using PSM analysis (Table S4). The cytokines that had statistical differences before PSM also had significant differences after PSM (Tables S5 and S6).

## 4 DISCUSSION

In recent years, many studies have conducted in‐depth exploration of the etiology and pathogenesis of AP. Although the causes of AP are diverse, they all have a common pathological process, namely the destruction of acinar cells and the abnormal activation of pancreatic protease induce the “self‐digestion” of the pancreas, which ultimately leads to the occurrence of AP.^19^ Damaged acinar cells release damage‐associated molecular patterns, which are recognized by pattern recognition receptors (PRRs), and PRRs activate tissue and peripheral blood leukocytes and trigger the release of inflammatory cytokines. Inflammatory mediators further secrete by infiltrating immune‐related cells, increase vascular permeability, cause neutrophil extravasation and activation, edema and microvascular disorder, and ultimately lead to hypoxia and tissue damage.^20^

The results of this study showed that the levels of seven out of 12 cytokines were significantly higher in the SAP group than in the mild or moderate AP group, namely IL‐1β, IL‐5, IL‐6, IL‐8, IL‐10, IL‐17, TNF‐α. Some studies have pointed out that among the many cytokines, TNF‐α is the earliest and most widely biologically effective cytokine, and also a key factor in causing SIRS and complications. Excessive TNF‐α enters the blood circulation, and stimulates the production of cytokines (IL‐1β, IL‐6, IL‐8), causing a “waterfall‐like cascade reaction,” leading to a cytokine storm. The correlation between disease severity and cytokine levels of IL‐1β, IL‐6, IL‐8, and IL‐10 has been repeatedly confirmed, but the sensitivity and specificity reported in different literature vary greatly.^21^, ^22^, ^23^ Sternby et al.^24^ performed paired comparisons of samples collected at 0–24 and 25–48 h after AP onset, and the results showed that IL‐1β, IL‐6, IL‐8, and IL‐10 changed over time, and there were significant differences between different severity groups after 24 h. Ćeranić et al.^25^ monitored the serum concentrations of IL‐6, IL‐8, and IL‐10 at the admission of AP patients, and compared with IL‐8 and IL‐10, IL‐6 had the highest predictive value for SAP at admission, which is consistent with our study.

In this study, we compared the cytokine differences between the ANC group and the non‐ANC group, and IL‐6, IL‐8, and IL‐10 showed good predictive value for the occurrence of ANC. In Kostic et al.'s^26^ study, the levels of IL‐6, IL‐8, and IL‐10 were significantly elevated in the necrosis group in the first 3 days of AP, which is similar to our study results. In addition, Schneider et al.^27^ studied the kinetics of inflammatory mediator release in necrotizing pancreatitis, and pancreatic injury increased continuously during the observation period, IL‐10 showed an early peak, and TNF‐α, and IL‐1β reached peaks at 6 and 9 h, respectively. In addition, in a prospective study of ICU patients with symptoms 48–72 h, IL‐1β levels were found to predict pancreatic necrosis with an accuracy of 88%.^28^ However, in this study, IL‐1β and TNF‐α did not show good predictive value for the occurrence of ANC. Unfortunately, we noticed that there was little previous prediction for APFC, and in our study, only IL‐6 showed predictive value among the 12 cytokines. Such results may be due to the fact that AP patients with AFPC have a milder local pancreatic inflammatory response.

It has been reported that the average time of occurrence of pleural effusion is about 4 days after AP onset.^29^ In this study, we also evaluated the availability of 12 serum cytokines within 48 h of symptom onset to predict AP patients with pleural effusion, and IL‐1β, IL‐6, IL‐8, and IL‐10 all showed good predictive value. Some studies have reported that AP patients with acute lung injury had significantly higher levels of IL‐1β, IL‐6, IL‐8, and TNF‐α.^30^ However, in this study, TNF‐α did not show value in predicting AP with pleural effusion. In addition, Vasseur et al.^31^ found that IL‐1β and IL‐10 were elevated in AP patients with pleural effusion. In a mouse lung model of acute necrotizing pancreatitis, IL‐6 and IL‐10 expression increased.^32^ At the end of this study, we evaluated the predictive value of 12 cytokines for AP patients with ascites, and IL‐6, IL‐8, and IL‐10 still had good value. However, there have been few studies reported on the relationship between cytokines and AP with ascites.

It is noteworthy that in our study, IL‐6 had a significant correlation with both SAP and the four complications. In 1973, IL‐6 was identified as a soluble factor secreted by T cells, which is important for B cells to produce antibodies.^33^ In experimental AP, the peak elevation of IL‐6 concentration in the systemic circulation was proven to be an early event before severe pancreatic injury.^34^ Since its discovery more than 50 years ago, the IL‐6 pathway has been confirmed as a key pathway involved in many disease disorders. Since the approval of anti‐IL‐6 receptor therapy, it has been a decade, and this therapy is now used worldwide for various rheumatic diseases, Castleman's disease, and cytokine release syndrome and other diseases.^35^ It is well known that IL‐6 has a dual role, so there is a wide debate on its homeostatic and pathogenic roles in various immune diseases when considering IL‐6 as a therapeutic target.^36^ Previous studies have shown that in the early stage of SAP, IL‐6 can exert anti‐inflammatory effects through its classical pathway, and when the level of IL‐6 in the body is too high, it can exert inflammatory effects through the transduction pathway, and can expand the local pancreatic tissue damage to the lungs.^37^

Cytokines are important multieffect regulators of immune responses, which have proinflammatory and anti‐inflammatory properties, and can produce effective defense responses against invading pathogens. On the other hand, cytokines may interfere with immune responses and enhance inflammation. Blocking the activity of proinflammatory cytokines can promote the survival of sepsis animal models, however, this therapeutic strategy has not improved clinical outcomes.^38^

The pathological and biological characteristics of AP are tissue damage leading to immune dysregulation, and the severity of the disease is related to the degree of immune response. The imbalance of the body's inflammatory response regulation is an important cause of severe or even death in patients with pancreatitis. Later studies have shown that there are two problems with early blockade therapy: one is that there are many types of inflammatory factors in the body of AP patients, and various inflammatory factors constitute a complex interaction network, which are both balanced and complementary. The treatment method of inhibiting one of the inflammatory factors will cause other network members to increase compensatory, resulting in no therapeutic effect. Secondly, the proinflammatory/anti‐inflammatory response in the AP process is dynamic, and the inflammatory factors and inflammatory cells will also change under different conditions.^39^ The complexity of the immune response requires fine‐tuned treatment intervention, and balanced treatment of proinflammatory and anti‐inflammatory responses may be a hopeful treatment option for AP.^1^

In the past, there were relatively few studies on the relationship between serum cytokine levels and common complications of AP. The advantage of this study is that it systematically evaluated the correlation between SAP and AP in‐hospital complications and 12 serum cytokine levels. However, some limitations of this study cannot be ignored. The sample size of this study was limited, the number of severe patients was small, and there was potential bias. Further studies and confirmation are needed in large‐scale, multicenter prospective studies, and the clinical value of different etiologies of AP patients can be further studied in the future. Secondly, as this study was a retrospective study, the sample collection time was a single time point within 48 h after admission, and longitudinal monitoring at different time points in the future can better understand the dynamic changes of AP. Third, the data of this study was limited, and there was a lack of comparison with other clinical inflammatory indicators.

In conclusion, our study revealed that serum cytokine levels, including IL‐1β, L‐6, IL‐8, and IL‐10, may be related to the occurrence of early complications of AP. Among them, IL‐6 has a relatively good predictive value. For the current era of monoclonal antibodies and other targeted therapies, clarifying the upregulated cytokines in the course of AP may provide reference for the future direction of targeted therapy research. Cytokines, as a cheap, repeatable, and noninvasive systemic inflammatory marker, monitoring IL‐6 levels within 48 h of AP admission may help predict the clinical outcomes of these patients.

## AUTHOR CONTRIBUTIONS

**Jiaxin Yao**: Conceptualization; data curation; formal analysis; methodology; software; visualization; writing—original draft; writing—review and editing. **Shuangshuang Zhang**: Data curation; investigation; software; visualization; writing—original draft. **Fei Zhou**: Investigation; resources; validation. **Mengting Zhuang**: Data curation. **Sujuan Fei**: Conceptualization; data curation; funding acquisition; project administration; resources; supervision; writing—review and editing.

## CONFLICT OF INTEREST STATEMENT

The authors declare no conflicts of interest.

## ETHICS STATEMENT

The study was conducted in accordance with the principles of the Declaration of Helsinki and was approved by the local ethics committee (Clinical Research Professional Ethics Committee of the First Affiliated Hospital of Xuzhou Medical University, China). Approval number: XYFY2023‐KL083‐01.

## Supporting information

Supporting information.

Supporting information.

## Data Availability

The data that support the findings of this study are available from the corresponding author upon reasonable request.

## References

[1] Mederos MA , Reber HA , Girgis MD . Acute pancreatitis: a review. JAMA. 2021;325(4):382‐390. 10.1001/jama.2020.20317 33496779

[2] Chan KS , Shelat VG . Diagnosis, severity stratification and management of adult acute pancreatitis—current evidence and controversies. World J Gastrointest Surg. 2022;14(11):1179‐1197. 10.4240/wjgs.v14.i11.1179 36504520 PMC9727576

[3] Jin M , Bai X , Chen X , et al. A 16‐year trend of etiology in acute pancreatitis: the increasing proportion of hypertriglyceridemia‐associated acute pancreatitis and its adverse effect on prognosis. J Clin Lipidol. 2019;13(6):947‐953.e1. 10.1016/j.jacl.2019.09.005 31735687

[4] Gliem N , Ammer‐Herrmenau C , Ellenrieder V , Neesse A . Management of severe acute pancreatitis: an update. Digestion. 2021;102(4):503‐507. 10.1159/000506830 32422634 PMC8315686

[5] Boxhoorn L , Voermans RP , Bouwense SA , et al. Acute pancreatitis. Lancet. 2020;396(10252):726‐734. 10.1016/S0140-6736(20)31310-6 32891214

[6] Luiken I , Eisenmann S , Garbe J , et al. Pleuropulmonary pathologies in the early phase of acute pancreatitis correlate with disease severity. PLoS One. 2022;17(2):e0263739. 10.1371/journal.pone.0263739 35130290 PMC8820650

[7] Zeng T , An J , Wu Y , et al. Incidence and prognostic role of pleural effusion in patients with acute pancreatitis: a meta‐analysis. Ann Med. 2023;55(2):2285909. 10.1080/07853890.2023.2285909 38010411 PMC10880572

[8] Karki A , Riley L , Mehta HJ , Ataya A . Abdominal etiologies of pleural effusion. Dis Mon. 2019;65(4):95‐103. 10.1016/j.disamonth.2018.09.001 30274930

[9] Bush N , Rana SS . Ascites in acute pancreatitis: clinical implications and management. Dig Dis Sci. 2022;67(6):1987‐1993. 10.1007/s10620-021-07063-6 34036465

[10] Komara NL , Paragomi P , Greer PJ , et al. Severe acute pancreatitis: capillary permeability model linking systemic inflammation to multiorgan failure. Am J Physiol Gastrointest Liver Physiol. 2020;319(5):G573‐G583. 10.1152/ajpgi.00285.2020 32877220 PMC8087347

[11] Siebert M , Le Fouler A , Sitbon N , Cohen J , Abba J , Poupardin E . Management of abdominal compartment syndrome in acute pancreatitis. J Visc Surg. 2021;158(5):411‐419. 10.1016/j.jviscsurg.2021.01.001 33516625

[12] Szatmary P , Grammatikopoulos T , Cai W , et al. Acute pancreatitis: diagnosis and treatment. Drugs. 2022;82(12):1251‐1276. 10.1007/s40265-022-01766-4 36074322 PMC9454414

[13] Mancilla Asencio C , Berger Fleiszig Z . Intra‐abdominal hypertension: a systemic complication of severe acute pancreatitis. Medicina. 2022;58(6):785. 10.3390/medicina58060785 35744049 PMC9229825

[14] Silva‐Vaz P , Abrantes AM , Castelo‐Branco M , Gouveia A , Botelho MF , Tralhão JG . Multifactorial scores and biomarkers of prognosis of acute pancreatitis: applications to research and practice. Int J Mol Sci. 2020;21(1):338. 10.3390/ijms21010338 31947993 PMC6982212

[15] Iyer S , Bawa EP , Tarique M , Dudeja V . Know thy enemy‐understanding the role of inflammation in severe acute pancreatitis. Gastroenterology. 2020;158(1):46‐48. 10.1053/j.gastro.2019.11.039 31770524

[16] Glaubitz J , Wilden A , Frost F , et al. Activated regulatory T‐cells promote duodenal bacterial translocation into necrotic areas in severe acute pancreatitis. Gut. 2023;72(7):1355‐1369. 10.1136/gutjnl-2022-327448 36631247 PMC10314084

[17] Wu J , Zhang L , Shi J , et al. Macrophage phenotypic switch orchestrates the inflammation and repair/regeneration following acute pancreatitis injury. EBioMedicine. 2020;58:102920. 10.1016/j.ebiom.2020.102920 32739869 PMC7399125

[18] Liu S , Szatmary P , Lin J , et al. Circulating monocytes in acute pancreatitis. Front Immunol. 2022;13:1062849. 10.3389/fimmu.2022.1062849 36578487 PMC9791207

[19] Zerem E , Kurtcehajic A , Kunosić S , Zerem Malkočević D , Zerem O . Current trends in acute pancreatitis: diagnostic and therapeutic challenges. World J Gastroenterol. 2023;29(18):2747‐2763. 10.3748/wjg.v29.i18.2747 37274068 PMC10237108

[20] Venkatesh K , Glenn H , Delaney A , Andersen CR , Sasson SC . Fire in the belly: a scoping review of the immunopathological mechanisms of acute pancreatitis. Front Immunol. 2022;13:1077414. 10.3389/fimmu.2022.1077414 36713404 PMC9874226

[21] Cho IR , Do MY , Han SY , Jang SI , Cho JH . Comparison of interleukin‐6, C‐reactive protein, procalcitonin, and the computed tomography severity index for early prediction of severity of acute pancreatitis. Gut Liver. 2024;17(4):629‐637. 10.5009/gnl220356 PMC10352050 36789576

[22] Tian F , Tian F , Tian F , et al. Correlation between severity of illness and levels of free triiodothyronine, interleukin‐6, and interleukin‐10 in patients with acute pancreatitis. Med Sci Monit. 2022;28:e933230. 10.12659/MSM.933230 35067670 PMC8796505

[23] Rodriguez‐Nicolas A , Martínez‐Chamorro A , Jiménez P , Matas‐Cobos AM , Redondo‐Cerezo E , Ruiz‐Cabello F . TH1 and TH2 cytokine profiles as predictors of severity in acute pancreatitis. Pancreas. 2018;47(4):400‐405. 10.1097/MPA.0000000000001006 29517628

[24] Sternby H , Hartman H , Thorlacius H , Regnér S . The initial course of IL1β, IL‐6, IL‐8, IL‐10, IL‐12, IFN‐γ and TNF‐α with regard to severity grade in acute pancreatitis. Biomolecules. 2021;11(4):591. 10.3390/biom11040591 33920566 PMC8073083

[25] Ćeranić DB , Zorman M , Skok P . Interleukins and inflammatory markers are useful in predicting the severity of acute pancreatitis. Bosn J Basic Med Sci. 2020;20(1):99‐105. 10.17305/bjbms.2019.4253 31242405 PMC7029213

[26] Kostic I , Spasic M , Stojanovic B , et al. Early cytokine profile changes in interstitial and necrotic forms of acute pancreatitis. Exp Appl Biomed Res. 2015;16(1):33‐37. 10.1515/sjecr-2015-0005

[27] Schneider L , Jabrailova B , Strobel O , Hackert T , Werner J . Inflammatory profiling of early experimental necrotizing pancreatitis. Life Sci. 2015;126:76‐80. 10.1016/j.lfs.2015.01.029 25711429

[28] Heresbach D , Letourneur JP , Bahon I , et al. Value of early blood Th‐1 cytokine determination in predicting severity of acute pancreatitis. Scand J Gastroenterol. 1998;33(5):554‐560. 10.1080/00365529850172160 9648999

[29] Polyzogopoulou E , Bikas C , Danikas D , Koutras A , Kalfarentzos F , Gogos CA . Baseline hypoxemia as a prognostic marker for pulmonary complications and outcome in patients with acute pancreatitis. Dig Dis Sci. 2004;49(1):150‐154. 10.1023/b:ddas.0000011617.00308.e3 14992450

[30] Samanta J , Singh S , Arora S , et al. Cytokine profile in prediction of acute lung injury in patients with acute pancreatitis. Pancreatology. 2018;18(8):878‐884. 10.1016/j.pan.2018.10.006 30361069

[31] Vasseur P , Devaure I , Sellier J , et al. High plasma levels of the pro‐inflammatory cytokine IL‐22 and the anti‐inflammatory cytokines IL‐10 and IL‐1ra in acute pancreatitis. Pancreatology. 2014;14(6):465‐469. 10.1016/j.pan.2014.08.005 25240697

[32] Vrolyk V , Schneberger D , Le K , Wobeser BK , Singh B . Mouse model to study pulmonary intravascular macrophage recruitment and lung inflammation in acute necrotizing pancreatitis. Cell Tissue Res. 2019;378(1):97‐111. 10.1007/s00441-019-03023-9 31037357

[33] Kishimoto T , Ishizaka K . Regulation of antibody response in vitro. J Immunol. 1973;111(4):1194‐1205. 4728681

[34] Czakó L , Takács T , Varga IS , et al. The pathogenesis of l‐arginine‐induced acute necrotizing pancreatitis: inflammatory mediators and endogenous cholecystokinin. J Physiol. 2000;94(1):43‐50. 10.1016/s0928-4257(99)00104-7 10761688

[35] Choy EH , De Benedetti F , Takeuchi T , Hashizume M , John MR , Kishimoto T . Translating IL‐6 biology into effective treatments. Nat Rev Rheumatol. 2020;16(6):335‐345. 10.1038/s41584-020-0419-z 32327746 PMC7178926

[36] Garbers C , Heink S , Korn T , Rose‐John S . Interleukin‐6: designing specific therapeutics for a complex cytokine. Nat Rev Drug Discov. 2018;17(6):395‐412. 10.1038/nrd.2018.45 29725131

[37] Zhang H , Neuhöfer P , Song L , et al. IL‐6 trans‐signaling promotes pancreatitis‐associated lung injury and lethality. J Clin Invest. 2013;123(3):1019‐1031. 10.1172/JCI64931 23426178 PMC3582130

[38] Zheng L , Xue J , Jaffee EM , Habtezion A . Role of immune cells and immune‐based therapies in pancreatitis and pancreatic ductal adenocarcinoma. Gastroenterology. 2013;144(6):1230‐1240. 10.1053/j.gastro.2012.12.042 23622132 PMC3641650

[39] Glaubitz J , Asgarbeik S , Lange R , et al. Immune response mechanisms in acute and chronic pancreatitis: strategies for therapeutic intervention. Front Immunol. 2023;14:1279539. 10.3389/fimmu.2023.1279539 37881430 PMC10595029

