# Supplementary material for: The relationship between inflammatory cytokines and in‐hospital complications of acute pancreatitis
Source: Immun Inflamm Dis. 2024 Feb 27;12(2):e1203. doi: 10.1002/iid3.1203 (PMC10898203; doi:10.1002/iid3.1203)
Supplement: Supplementary file 2 — Supporting information. [file IID3-12-e1203-s002.docx]

Table S1 Logistic regression analyses for the associations between the serum cytokine levels and the presence of ANC and APFC

| Variables | Model1 | | Model2 | | Model3 | |
| --- | --- | --- | --- | --- | --- | --- |
|  | OR/cOR(95% CI) | *p* value | OR/cOR(95% CI) | *p* value | OR/cOR(95% CI) | *p* value |
| **ANC** |  |  |  |  |  |  |
| IL-6 |  |  |  |  |  |  |
| Continuous (per 1-SD augment) | 1.34(1.05-1.71) | <0.05 | 1.34(1.05-1.71) | <0.05 | 1.33(1.04-1.71) | <0.05 |
| Q1 (≤4.47) | Ref. |  | Ref. |  | Ref. |  |
| Q2 (4.48-15.18) | 1.41(0.68-2.93) | 0.36 | 1.40(0.68-2.92) | 0.37 | 1.47(0.70-3.08) | 0.31 |
| Q3 (15.19-48.91) | 3.62(1.80-7.29) | <0.001 | 3.65(1.81-7.37) | <0.001 | 3.99(1.95-8.16) | <0.001 |
| Q4 (≥48.92) | 4.13(2.05-8.35) | <0.001 | 4.14(2.05-8.37) | <0.001 | 4.32(2.12-8.82) | <0.001 |
| IL-8 |  |  |  |  |  |  |
| Continuous (per 1-SD augment) | 1.65(1.13-2.41) | <0.05 | 1.64(1.13-2.39) | <0.05 | 1.66(1.13-2.45) | <0.05 |
| Q1 (≤2.30) | Ref. |  | Ref. |  | Ref. |  |
| Q2 (2.31-6.72) | 0.52(0.25-1.06) | 0.07 | 0.51(0.25-1.06) | 0.07 | 0.53(0.25-1.10) | 0.09 |
| Q3 (6.73-19.53) | 1.64(0.85-3.14) | 0.14 | 1.64(0.85-3.16) | 0.14 | 1.68(0.87-3.24) | 0.13 |
| Q4 (≥19.54) | 2.42(1.26-4.66) | <0.01 | 2.42(1.26-4.68) | <0.01 | 2.47(1.27-4.84) | <0.01 |
| IL-10 |  |  |  |  |  |  |
| Continuous (per 1-SD augment) | 1.32(0.94-1.87) | 0.11 | 1.32(0.93-1.88) | 0.12 | 1.33(0.94-1.88) | 0.11 |
| Q1 (≤1.05) | Ref. |  | Ref. |  | Ref. |  |
| Q2 (1.06-1.75) | 2.13(1.05-4.33) | <0.05 | 2.13(1.05-4.35) | <0.05 | 2.22(1.09-4.56) | <0.05 |
| Q3 (1.76-3.38) | 3.27(1.62-6.57) | <0.01 | 3.24(1.60-6.55) | <0.01 | 3.36(1.64-6.89) | <0.01 |
| Q4 (≥3.39) | 3.18(1.57-6.41) | <0.01 | 3.18(1.57-6.42) | <0.01 | 3.19(1.57-6.51) | <0.01 |
| **APFC** |  |  |  |  |  |  |
| IL-6 |  |  |  |  |  |  |
| Continuous (per 1-SD augment) | 1.27(0.99-1.63) | 0.06 | 1.30(1.01-1.69) | <0.05 | 1.32(1.02-1.72) | <0.05 |
| Q1 (≤4.47) | Ref. |  | Ref. |  | Ref. |  |
| Q2 (4.48-15.18) | 2.40(0.79-7.27) | 0.12 | 2.51(0.82-7.71) | 0.11 | 2.50(0.81-7.73) | 0.11 |
| Q3 (15.19-48.91) | 4.08(1.42-11.71) | 0.01 | 4.31(1.48-12.60) | <0.01 | 4.33(1.47-12.72) | <0.01 |
| Q4 (≥48.92) | 4.15(1.45-11.92) | 0.01 | 4.82(1.64-14.15) | <0.01 | 4.77(1.62-14.06) | <0.01 |

Abbreviations: OR, odds ratio; cOR, common OR CI, confidence interval; ANC, acute necrotic collection; APFC, acute peripancreatic fluid collection.

Model 1: crude logistic regression analysis;

Model 2: adjusted for age, sex, and BMI;

Model 3: adjusted for age, sex, BMI, hypertension, diabetes mellitus, coronary heart disease, smoking, and drinking.

Table S2 Logistic regression analyses for the associations between the serum cytokine levels and the presence of pleural effusion

| Variables | Model1 | | Model2 | | Model3 | |
| --- | --- | --- | --- | --- | --- | --- |
|  | OR/cOR(95% CI) | *p* value | OR/cOR(95% CI) | *p* value | OR/cOR(95% CI) | *p* value |
| IL-1β |  |  |  |  |  |  |
| Continuous (per 1-SD augment) | 1.29(1.01-1.66) | <0.05 | 1.32(1.02-1.70) | <0.05 | 1.33(1.02-1.72) | <0.05 |
| Q1 (≤2.31) | Ref. |  | Ref. |  | Ref. |  |
| Q2 (2.32-5.00) | 0.91(0.47-1.74) | 0.77 | 0.91(0.46-1.79) | 0.79 | 0.91(0.46-1.80) | 0.79 |
| Q3 (5.01-13.36) | 1.30(0.68-2.49) | 0.43 | 1.26(0.65-2.46) | 0.49 | 1.27(0.65-2.48) | 0.49 |
| Q4 (≥13.37) | 2.28(1.91-4.35) | <0.05 | 2.32(1.20-4.50) | <0.05 | 2.36(1.21-4.58) | <0.05 |
| IL-6 |  |  |  |  |  |  |
| Continuous (per 1-SD augment) | 1.61(1.20-2.16) | <0.01 | 1.63(1.20-2.21) | <0.01 | 1.64(1.22-2.22) | <0.01 |
| Q1 (≤4.47) | Ref. |  | Ref. |  | Ref. |  |
| Q2 (4.48-15.18) | 1.70(0.83-3.48) | 0.15 | 1.66(0.80-3.43) | 0.17 | 1.71(0.82-3.56) | 0.15 |
| Q3 (15.19-48.91) | 4.24(2.10-8.54) | <0.001 | 4.57(2.24-9.32) | <0.001 | 4.67(2.27-9.61) | <0.001 |
| Q4 (≥48.92) | 6.79(3.31-13.91) | <0.001 | 7.14(3.44-14.84) | <0.001 | 7.37(3.52-15.43) | <0.001 |
| IL-8 |  |  |  |  |  |  |
| Continuous (per 1-SD augment) | 1.44(1.03-2.02) | <0.05 | 1.44(1.03-2.00) | <0.05 | 1.50(1.05-2.12) | <0.05 |
| Q1 (≤2.30) | Ref. |  | Ref. |  | Ref. |  |
| Q2 (2.31-6.72) | 1.13(0.57-2.21) | 0.73 | 1.12(0.57-2.22) | 0.74 | 1.10(0.55-2.18) | 0.79 |
| Q3 (6.73-19.53) | 3.11(1.60-6.02) | <0.01 | 2.98(1.52-5.81) | <0.01 | 2.95(1.51-5.79) | <0.01 |
| Q4 (≥19.54) | 2.33(1.20-4.50) | <0.05 | 2.26(1.16-4.40) | <0.05 | 2.31(1.17-4.55) | <0.05 |
| IL-10 |  |  |  |  |  |  |
| Continuous (per 1-SD augment) | 3.25(1.41-7.51) | <0.01 | 3.54(1.50-8.36) | <0.01 | 3.96(1.60-9.78) | <0.01 |
| Q1 (≤1.05) | Ref. |  | Ref. |  | Ref. |  |
| Q2 (1.06-1.75) | 1.57(0.81-3.04) | 0.18 | 1.50(0.77-2.93) | 0.23 | 1.51(0.77-2.97) | 0.23 |
| Q3 (1.76-3.38) | 1.84(0.95-3.56) | 0.07 | 1.72(0.88-3.35) | 0.11 | 1.79(0.91-3.55) | 0.09 |
| Q4 (≥3.39) | 2.88(1.48-5.58) | <0.01 | 2.99(1.53-5.86) | <0.01 | 3.20(1.61-6.36) | <0.01 |

Abbreviations: OR, odds ratio; cOR, common OR CI, confidence interval;

Model 1: crude logistic regression analysis;

Model 2: adjusted for age, sex, and BMI;

Model 3: adjusted for age, sex, BMI, hypertension, diabetes mellitus, coronary heart disease, smoking, and drinking.

Table S3 Logistic regression analyses for the associations between the serum cytokine levels and the presence of ascites

| Variables | Model1 | | Model2 | | Model3 | |
| --- | --- | --- | --- | --- | --- | --- |
|  | OR/cOR(95% CI) | *p* value | OR/cOR(95% CI) | *p* value | OR/cOR(95% CI) | *p* value |
| IL-1β |  |  |  |  |  |  |
| Continuous (per 1-SD augment) | 1.09(0.87-1.38) | 0.44 | 1.10(0.87-1.38) | 0.42 | 1.10(0.87-1.39) | 0.42 |
| Q1 (≤2.31) | Ref. |  | Ref. |  | Ref. |  |
| Q2 (2.32-5.00) | 1.15(0.59-2.27) | 0.68 | 1.19(0.59-2.40) | 0.62 | 1.23(0.60-2.51) | 0.57 |
| Q3 (5.01-13.36) | 1.17(0.59-2.33) | 0.65 | 1.22(0.61-2.45) | 0.58 | 1.22(0.60-2.48) | 0.58 |
| Q4 (≥13.37) | 1.62(0.83-3.16) | 0.16 | 1.67(0.85-3.28) | 0.14 | 1.76(0.88-3.49) | 0.11 |
| IL-6 |  |  |  |  |  |  |
| Continuous (per 1-SD augment) | 1.82(1.34-2.48) | <0.001 | 1.85(1.36-2.54) | <0.001 | 1.86(1.37-2.54) | <0.001 |
| Q1 (≤4.47) | Ref. |  | Ref. |  | Ref. |  |
| Q2 (4.48-15.18) | 1.45(0.68-3.10) | 0.34 | 1.44(0.67-3.09) | 0.35 | 1.58(0.73-3.45) | 0.25 |
| Q3 (15.19-48.91) | 2.64(1.28-5.46) | <0.01 | 2.77(1.33-5.76) | <0.01 | 3.01(1.42-6.37) | <0.01 |
| Q4 (≥48.92) | 4.59(2.23-9.45) | <0.001 | 4.81(2.32-9.97) | <0.001 | 5.06(2.40-10.64) | <0.001 |
| IL-8 |  |  |  |  |  |  |
| Continuous (per 1-SD augment) | 1.56(1.11-2.18) | <0.05 | 1.57(1.11-2.21) | <0.05 | 1.63(1.13-2.34) | <0.01 |
| Q1 (≤2.30) | Ref. |  | Ref. |  | Ref. |  |
| Q2 (2.31-6.72) | 0.72(0.35-1.47) | 0.36 | 0.71(0.35-1.46) | 0.35 | 0.66(0.32-1.38) | 0.27 |
| Q3 (6.73-19.53) | 1.86(0.96-3.61) | 0.07 | 1.80(0.92-3.51) | 0.09 | 1.73(0.88-3.42) | 0.11 |
| Q4 (≥19.54) | 1.53(0.78-2.99) | 0.21 | 1.58(0.80-3.10) | 0.19 | 1.49(0.75-2.99) | 0.26 |
| IL-10 |  |  |  |  |  |  |
| Continuous (per 1-SD augment) | 1.24(0.93-1.65) | 0.14 | 1.28(0.95-1.72) | 0.1 | 1.28(0.94-1.74) | 0.11 |
| Q1 (≤1.05) | Ref. |  | Ref. |  | Ref. |  |
| Q2 (1.06-1.75) | 1.80(0.88-3.68) | 0.11 | 1.73(0.84-3.56) | 0.14 | 1.82(0.88-3.80) | 0.11 |
| Q3 (1.76-3.38) | 2.25(1.11-4.57) | <0.05 | 2.26(1.11-4.61) | <0.05 | 2.57(1.23-5.37) | <0.05 |
| Q4 (≥3.39) | 2.57(1.27-5.20) | <0.01 | 2.61(1.29-5.31) | <0.01 | 2.84(1.37-5.90) | <0.01 |

Abbreviations: OR, odds ratio; cOR, common OR CI, confidence interval;

Model 1: crude logistic regression analysis;

Model 2: adjusted for age, sex, and BMI;

Model 3: adjusted for age, sex, BMI, hypertension, diabetes mellitus, coronary heart disease, smoking, and drinking.

Table S4 Comparison of Baseline Characteristics Before and After PSM between the complication group and non-complication group

|  | Pre-PS March | | *p* value | Post-PS March | | *p* value |
| --- | --- | --- | --- | --- | --- | --- |
| ANC | Complication  (n=119) | No Complication  (n=188) |  | Complication  (n=114) | No Complication  (n=166) |  |
| Age, median (IQR), years | 45(33,67) | 45(34,59.75) | 0.923 | 45(33,66.25) | 47.5(34.75,60.5) | 0.551 |
| Gender, n (%) | 56(47.1) | 88(46.8) | 0.966 | 53(46.5) | 72(43.4) | 0.606 |
| BMI, median (IQR) | 25.95(24.14,27.06) | 26.22(23.62,28.73) | 0.426 | 26.04(24.20,27.27) | 26.08(23.34,28.73) | 0.754 |
| Hypertension, n (%) | 36(30.3) | 56(29.8) | 0.931 | 34(29.8) | 47(28.3) | 0.784 |
| Diabetes mellitus, n (%) | 35(29.4) | 48(25.5) | 0.456 | 31(27.2) | 38(22.9) | 0.412 |
| Coronary heart disease, n (%) | 13(10.9) | 22(11.7) | 0.835 | 12(10.5) | 17(10.2) | 0.939 |
| smoking, n (%) | 25(21) | 48(25.5) | 0.364 | 25(21.9) | 36(21.7) | 0.961 |
| drinking, n (%) | 17(14.3） | 29(15.4) | 0.785 | 17(14.9) | 24(14.5) | 0.916 |
|  | Pre-PS March | |  | Post-PS March | |  |
| APFC | Complication  (n=50) | No Complication  (n=257) |  | Complication  (n=50) | No Complication  (n=95) |  |
| Age, median (IQR), years | 37(31.75,42.25) | 49(34.5,65) | 0.001 | 37(31.75,45.25) | 36(32,47) | 0.792 |
| Gender, n (%) | 24(48) | 120(46.7) | 0.865 | 24(48) | 44(46.3) | 0.847 |
| BMI, median (IQR) | 26.22(24.13,29.57) | 26.09(23.86,27.99) | 0.598 | 26.22(24.13,29.57) | 26.22(24.22,29.41) | 0.706 |
| Hypertension, n (%) | 10(20) | 82(31.9) | 0.093 | 10(20) | 22(23.2) | 0.663 |
| Diabetes mellitus, n (%) | 13(26) | 70(27.2) | 0.857 | 13(26) | 30(31.6) | 0.484 |
| Coronary heart disease, n (%) | 4(8) | 31(12.1) | 0.408 | 4(8) | 8(8.4) | 0.93 |
| smoking, n (%) | 14(28) | 59(23) | 0.443 | 14(28) | 26(27.4) | 0.936 |
| drinking, n (%) | 8(16) | 38(14.8) | 0.826 | 8(16) | 17(17.9) | 0.744 |
|  | Pre-PS March | |  | Post-PS March | |  |
| Pleural Effusion | Complication  (n=134) | No Complication  (n=173) |  | Complication  (n=130) | No Complication  (n=164) |  |
| Age, median (IQR), years | 46(33.75,66) | 44(34,60) | 0.323 | 45.5(33,65.25) | 45(34.25,60) | 0.874 |
| Gender, n (%) | 57(42.5) | 87(50.3) | 0.177 | 57(43.8) | 79(48.2) | 0.46 |
| BMI, median (IQR) | 25.99(23.80,26.93) | 26.22(23.99,28.74) | 0.14 | 26.08(24.08,27.05) | 26(23.85,28.40) | 0.604 |
| Hypertension, n (%) | 41(30.6) | 51(29.5) | 0.832 | 39(30) | 51(31.1) | 0.839 |
| Diabetes mellitus, n (%) | 31(23.1) | 52(30.1) | 0.176 | 31(23.8) | 43(26.2) | 0.641 |
| Coronary heart disease, n (%) | 14(10.4) | 21(12.1) | 0.644 | 14(10.8) | 17(10.4) | 0.911 |
| smoking, n (%) | 29(21.6) | 44(25.4) | 0.439 | 29(22.3) | 39(23.8) | 0.766 |
| drinking, n (%) | 19(14.2) | 27(15.6) | 0.728 | 19(14.6) | 25(15.2) | 0.881 |
|  | Pre-PS March | |  | Post-PS March | |  |
| Ascites | Complication  (n=105) | No Complication  (n=202) |  | Complication  (n=102) | No Complication  (n=174) |  |
| Age, median (IQR), years | 44(33.5,66) | 47(34,60) | 0.641 | 44(33.75,66) | 47(35,60) | 0.512 |
| Gender, n (%) | 44(41.9) | 100(49.5) | 0.206 | 43(42.2) | 76(43.7) | 0.805 |
| BMI, median (IQR) | 25.95(23.84,27.20) | 26.22(23.93,28.49) | 0.367 | 25.89(23.80,26.81) | 26.07(23.88,28.35) | 0.469 |
| Hypertension, n (%) | 30(28.6) | 62(30.7) | 0.7 | 29(28.4) | 53(30.5) | 0.722 |
| Diabetes mellitus, n (%) | 25(23.8) | 58(28.7) | 0.359 | 24(23.5) | 44(25.3) | 0.744 |
| Coronary heart disease, n (%) | 12(11.4) | 23(11.4) | 0.991 | 11(10.8) | 17(9.8) | 0.788 |
| smoking, n (%) | 19(18.1) | 54(26.7) | 0.092 | 18(17.6) | 30(17.2) | 0.932 |
| drinking, n (%) | 18(17.1) | 28(13.9) | 0.445 | 16(15.7) | 26(14.9) | 0.868 |

Abbreviations: ANC, acute necrotic collection; APFC, acute peripancreatic fluid collection; BMI, body mass index.

Table S5 The relationship between serum cytokine levels and the presence of ANC and APFC after propensity score matching

| Complication | Model 3 adjusted | |  | Post-PS March | |
| --- | --- | --- | --- | --- | --- |
|  | OR/cOR(95% CI) | *p* value |  | OR/cOR(95% CI) | *p* value |
| **ANC** |  |  | **ANC** |  |  |
| IL-6 |  |  | IL-6 |  |  |
| Continuous (per 1-SD augment) | 1.33(1.04-1.71) | <0.05 | Continuous (per 1-SD augment) | 1.30(1.01-1.67) | <0.05 |
| Q1 (≤4.47) | Ref. |  | Q1 (≤4.79) | Ref. |  |
| Q2 (4.48-15.18) | 1.47(0.70-3.08) | 0.31 | Q2 (4.80-16.19) | 1.34(0.63-2.82) | 0.45 |
| Q3 (15.19-48.91) | 3.99(1.95-8.16) | <0.001 | Q3 (16.20-49.14) | 3.50(1.70-7.18) | <0.01 |
| Q4 (≥48.92) | 4.32(2.12-8.82) | <0.001 | Q4 (≥49.15) | 3.92(1.91-8.07) | <0.001 |
| IL-8 |  |  | IL-8 |  |  |
| Continuous (per 1-SD augment) | 1.66(1.13-2.45) | <0.05 | Continuous (per 1-SD augment) | 1.81(1.12-2.93) | <0.05 |
| Q1 (≤2.30) | Ref. |  | Q1 (≤2.33) | Ref. |  |
| Q2 (2.31-6.72) | 0.53(0.25-1.10) | 0.09 | Q2 (2.34-6.73) | 0.53(0.25-1.12) | 0.1 |
| Q3 (6.73-19.53) | 1.68(0.87-3.24) | 0.13 | Q3 (6.74-19.31) | 1.70(0.86-3.35) | 0.13 |
| Q4 (≥19.54) | 2.47(1.27-4.84) | <0.01 | Q4 (≥19.32) | 2.27(1.15-4.47) | <0.05 |
| IL-10 |  |  | IL-10 |  |  |
| Continuous (per 1-SD augment) | 1.33(0.94-1.88) | 0.11 | Continuous (per 1-SD augment) | 1.23(0.89-1.70) | 0.2 |
| Q1 (≤1.05) | Ref. |  | Q1 (≤1.11) | Ref. |  |
| Q2 (1.06-1.75) | 2.22(1.09-4.56) | <0.05 | Q2 (1.12-1.76) | 2.37(1.14-4.90) | <0.05 |
| Q3 (1.76-3.38) | 3.36(1.64-6.89) | <0.01 | Q3 (1.77-3.29) | 3.29(1.61-6.75) | <0.01 |
| Q4 (≥3.39) | 3.19(1.57-6.51) | <0.01 | Q4 (≥3.30) | 3.11(1.52-6.37) | <0.01 |
| **APFC** |  |  | **APFC** |  |  |
| IL-6 |  |  | IL-6 |  |  |
| Continuous (per 1-SD augment) | 1.32(1.02-1.72) | <0.05 | Continuous (per 1-SD augment) | 1.33(0.94-1.89) | 0.11 |
| Q1 (≤4.47) | Ref. |  | Q1 (≤6.17) | Ref. |  |
| Q2 (4.48-15.18) | 2.50(0.81-7.73) | 0.11 | Q2 (6.18-22.77) | 2.14(0.73-6.28) | 0.17 |
| Q3 (15.19-48.91) | 4.33(1.47-12.72) | <0.01 | Q3 (22.78-55.48) | 2.73(0.94-7.88) | 0.06 |
| Q4 (≥48.92) | 4.77(1.62-14.06) | <0.01 | Q4 (≥55.49) | 3.84(1.34-10.97) | <0.05 |

Abbreviations: ANC, acute necrotic collection; APFC, acute peripancreatic fluid collection.

Table S6 The relationship between serum cytokine levels and the presence of Pleural effusion and ascites after propensity score matching

| Complication | Model 3 adjusted | |  | Post-PS March | |
| --- | --- | --- | --- | --- | --- |
|  | OR/cOR(95% CI) | *p* value |  | OR/cOR(95% CI) | *p* value |
| **Pleural effusion** |  |  | **Pleural effusion** |  |  |
| IL-1β |  |  | IL-1β |  |  |
| Continuous (per 1-SD augment) | 1.33(1.02-1.72) | <0.05 | Continuous (per 1-SD augment) | 1.36(1.04-1.78) | <0.05 |
| Q1 (≤2.31) | Ref. |  | Q1 (≤2.31) | Ref. |  |
| Q2 (2.32-5.00) | 0.91(0.46-1.80) | 0.79 | Q2 (2.32-4.97) | 1.08(0.56-2.11) | 0.82 |
| Q3 (5.01-13.36) | 1.27(0.65-2.48) | 0.49 | Q3 (4.98-12.87) | 1.19(0.61-2.30) | 0.61 |
| Q4 (≥13.37) | 2.36(1.21-4.58) | <0.05 | Q4 (≥12.88) | 2.80(1.43-5.46) | <0.01 |
| IL-6 |  |  | IL-6 |  |  |
| Continuous (per 1-SD augment) | 1.64(1.22-2.22) | <0.01 | Continuous (per 1-SD augment) | 1.66(1.21-2.27) | <0.01 |
| Q1 (≤4.47) | Ref. |  | Q1 (≤4.39) | Ref. |  |
| Q2 (4.48-15.18) | 1.71(0.82-3.56) | 0.15 | Q2 (4.40-14.69) | 1.54(0.74-3.21) | 0.25 |
| Q3 (15.19-48.91) | 4.67(2.27-9.61) | <0.001 | Q3 (14.70-47.32) | 4.40(2.16-8.96) | <0.001 |
| Q4 (≥48.92) | 7.37(3.52-15.43) | <0.001 | Q4 (≥47.33) | 6.44(3.12-13.31) | <0.001 |
| IL-8 |  |  | IL-8 |  |  |
| Continuous (per 1-SD augment) | 1.50(1.05-2.12) | <0.05 | Continuous (per 1-SD augment) | 1.46(1.03-2.05) | <0.05 |
| Q1 (≤2.30) | Ref. |  | Q1 (≤2.22) | Ref. |  |
| Q2 (2.31-6.72) | 1.10(0.55-2.18) | 0.79 | Q2 (2.23-6.61) | 1.09(0.55-2.16) | 0.82 |
| Q3 (6.73-19.53) | 2.95(1.51-5.79) | <0.01 | Q3 (6.62-19.53) | 2.89(1.48-5.65) | <0.01 |
| Q4 (≥19.54) | 2.31(1.17-4.55) | <0.05 | Q4 (≥19.54) | 2.26(1.16-4.42) | <0.05 |
| IL-10 |  |  | IL-10 |  |  |
| Continuous (per 1-SD augment) | 3.96(1.60-9.78) | <0.01 | Continuous (per 1-SD augment) | 3.59(1.46-8.84) | <0.01 |
| Q1 (≤1.05) | Ref. |  | Q1 (≤1.05) | Ref. |  |
| Q2 (1.06-1.75) | 1.51(0.77-2.97) | 0.23 | Q2 (1.06-1.74) | 1.64(0.83-3.22) | 0.15 |
| Q3 (1.76-3.38) | 1.79(0.91-3.55) | 0.09 | Q3 (1.75-3.22) | 1.69(0.86-3.31) | 0.13 |
| Q4 (≥3.39) | 3.20(1.61-6.36) | <0.01 | Q4 (≥3.23) | 3.36(1.71-6.64) | <0.001 |
| **Ascites** |  |  | **Ascites** |  |  |
| IL-6 |  |  | IL-6 |  |  |
| Continuous (per 1-SD augment) | 1.86(1.37-2.54) | <0.001 | Continuous (per 1-SD augment) | 1.83(1.32-2.53) | <0.001 |
| Q1 (≤4.47) | Ref. |  | Q1 (≤4.79) | Ref. |  |
| Q2 (4.48-15.18) | 1.58(0.73-3.45) | 0.25 | Q2 (4.80-16.15) | 1.49(0.68-3.29) | 0.32 |
| Q3 (15.19-48.91) | 3.01(1.42-6.37) | <0.01 | Q3 (16.16-50.96) | 3.40(1.60-7.22) | <0.01 |
| Q4 (≥48.92) | 5.06(2.40-10.64) | <0.001 | Q4 (≥50.97) | 4.54(2.14-9.65) | <0.001 |
| IL-8 |  |  | IL-8 |  |  |
| Continuous (per 1-SD augment) | 1.63(1.13-2.34) | <0.01 | Continuous (per 1-SD augment) | 1.56(1.09-2.22) | <0.05 |
| Q1 (≤2.30) | Ref. |  | Q1 (≤2.26) | Ref. |  |
| Q2 (2.31-6.72) | 0.66(0.32-1.38) | 0.27 | Q2 (2.27-6.85) | 0.81(0.39-1.69) | 0.58 |
| Q3 (6.73-19.53) | 1.73(0.88-3.42) | 0.11 | Q3 (6.86-21.00) | 1.74(0.87-3.49) | 0.12 |
| Q4 (≥19.54) | 1.49(0.75-2.99) | 0.26 | Q4 (≥21.01) | 1.64(0.82-3.29) | 0.16 |
| IL-10 |  |  | IL-10 |  |  |
| Continuous (per 1-SD augment) | 1.28(0.94-1.74) | 0.11 | Continuous (per 1-SD augment) | 1.24(0.92-1.68) | 0.16 |
| Q1 (≤1.05) | Ref. |  | Q1 (≤1.10) | Ref. |  |
| Q2 (1.06-1.75) | 1.82(0.88-3.80) | 0.11 | Q2 (1.11-1.78) | 2.00(0.95-4.20) | 0.07 |
| Q3 (1.76-3.38) | 2.57(1.23-5.37) | <0.05 | Q3 (1.79-3.43) | 2.26(1.08-4.73) | <0.05 |
| Q4 (≥3.39) | 2.84(1.37-5.90) | <0.01 | Q4 (≥3.44) | 2.87(1.38-5.96) | <0.01 |


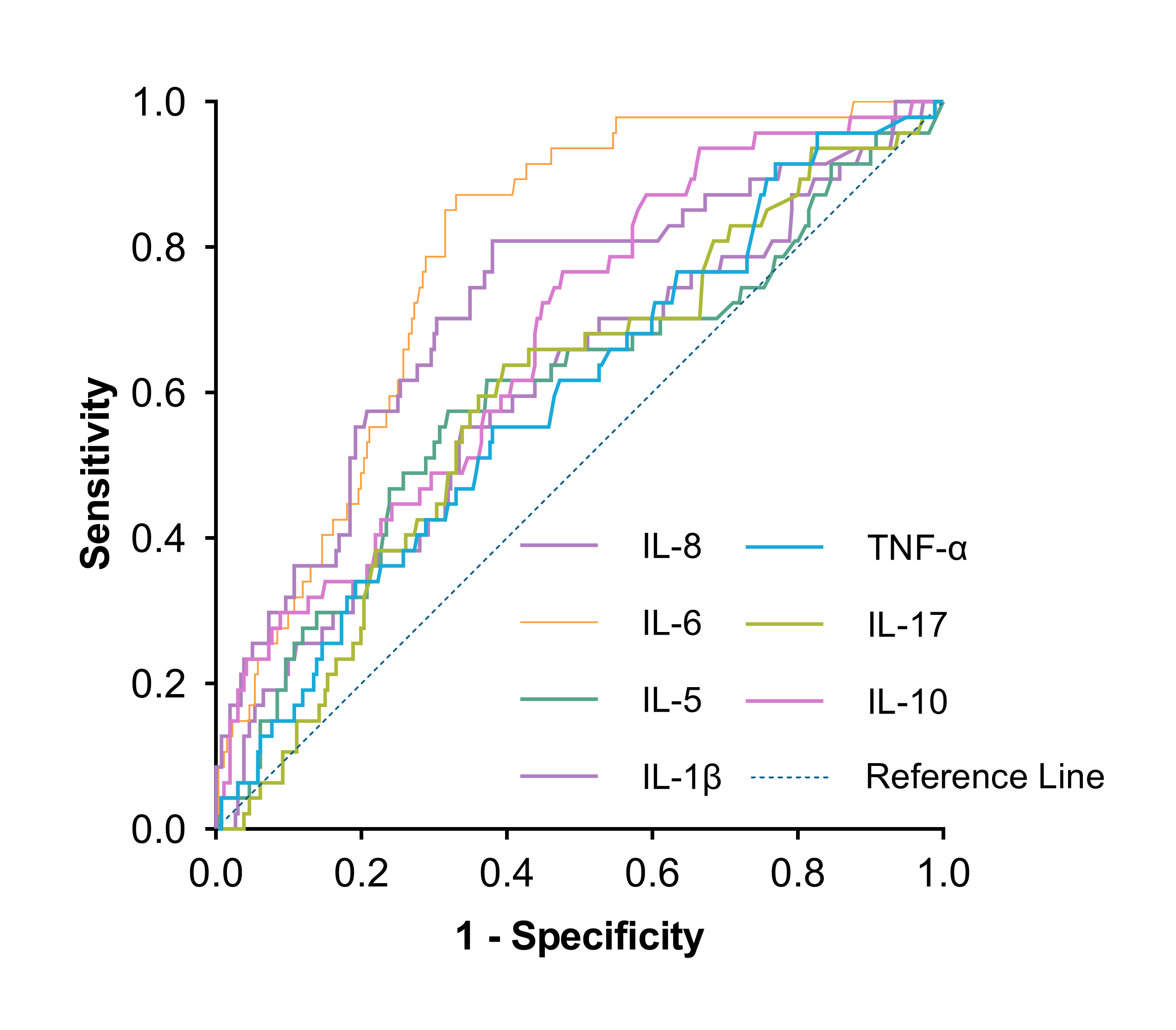


Figure S1 Receiver Operating Characteristic Curves of Cytokines for Prediction of SAP.

Abbreviations: IL-1β, interleukin-1βeta; IL-5, interleukin-5; IL-6, interleukin-6; IL-8, interleukin-8; IL-10,interleukin-10; IL-17, interleukin-17; TNF-α, tumor necrosis factor-alpha.
